# Deterioration of Health-Related Quality of Life Scores under Treatment Predicts Longer Survival

**DOI:** 10.1155/2020/3565238

**Published:** 2020-08-17

**Authors:** Maike Jörling, Sandra Rutzner, Markus Hecht, Rainer Fietkau, Luitpold V. Distel

**Affiliations:** Department of Radiation Oncology, Universitätsklinikum Erlangen, Friedrich-Alexander-Universität Erlangen-Nürnberg, 91054 Erlangen, Germany

## Abstract

**Objectives:**

Baseline health-related quality of life (HRQoL) scores predict survival, which has already been demonstrated in various studies. However, we were interested in whether changes in baseline scores during treatment are also significant predictors of survival.

**Methods and Materials:**

We analysed the data of 400 consecutive cancer patients receiving radiochemotherapy. Leading diagnoses were head and neck cancer (34.5%), rectal cancer (24.5%), and lung cancer (13%). HRQoL was studied at baseline, six weeks after therapy and after each completed year after the start of therapy until drop out of the study using the EORTC QLQ-C30 questionnaire. The change score was calculated as the baseline score subtracted from the score after therapy. Statistics included Kaplan-Meier estimates and Cox regression.

**Results:**

High global health status (*p* = 0.005) and low pain scores (*p* = 0.040) at baseline were related to favourable overall survival. Change scores of role functioning (*p* = 0.027), global health status (*p* < 0.018), and pain (*p* < 0.001) were predictive of overall survival. Pain was the superior predictor of survival (*p* = 0.001) among all variables and QoL scores studied by multivariate analysis. A deterioration in pain was associated with a 2.8 times higher chance of survival (HR 0.36).

**Conclusions:**

Deterioration of HRQoL baseline pain score by cancer treatment is a favourable and superior prognostic factor for survival.

## 1. Introduction

Great scientific progress has been achieved in the fields of medicine, hygiene, and health care in recent decades, resulting in prolonged survival times for patients [1]. However, the number of cancer patients will further increase due to demographic change [2]. Both aspects together have already resulted in a dramatically increased number of long-term survivors of cancer and it will continue to rise [3, 4]. Another factor influencing this is increasingly specialized therapy options [5, 6].

Statistics from the USA prove that cancer plays an important role; in 2016, cancer was the second most frequent cause of death in the United States and one in four deaths could be attributed to cancer [7]. Important influencing and risk factors in recent decades such as smoking have also led to cancer cases becoming more frequent and therapy becoming increasingly important. In the last several years, scientists have discovered that successful therapy and survival are closely related to quality of life.

Cancer patients experience a wide variety of symptoms and reach their limits when trying to manage them. Their symptoms directly influence their quality of life. Therefore, symptom management improves QoL [8, 9]. Although more research has been done in recent years regarding these topics, this is not yet sufficient. As the number of cancer survivors continues to rise, not only the pure survival time but also the late effects of radiochemotherapy and the health-related quality of life (HRQoL) are crucial for patients. Radiochemotherapy in particular has a major influence on HRQoL [10]. HRQoL is highly relevant for patient care; it directly influences therapy, satisfaction, and compliance. Since patients themselves are the best source for measuring HRQoL, various questionnaires are used in clinical trials worldwide. HRQoL is important for making treatment decisions; especially, the questionnaire QLQ-C30 of the EORTC is recommended as a measuring instrument [11]. In particular, there is too little research on whether a poor HRQoL consequently means shorter survival time.

Some studies already indicated that the baseline score predicts overall survival (OS), both for a heterogeneous group of cancer types [12–16] and for certain tumour types, e.g., for lung cancer [17–21], head and neck cancer [22–24] and colorectal cancer [25, 26]. In addition, it has been suggested that older patients are less affected by radiochemotherapeutic treatment than younger patients [27].

However, it is important to know whether one can deduce survival from a certain factor. Furthermore, only few studies have addressed whether a change in HRQoL between before and after therapy is decisive for survival [21, 25, 28]. The general assumption is that patients who tolerate therapy better or recover faster will survive longer. But is this the case? For this reason, our aim was to investigate how HRQoL before therapy, after therapy, and the changes in between those two points in time affect survival.

## 2. Patients and Methods

### 2.1. Study Population

This clinical trial included 400 consecutive patients who met the inclusion criteria. These were a clinically diagnosed cancer treated with radiochemotherapy in our clinic and the ability to understand and complete the EORTC QLQ-C30 questionnaire. Patients were surveyed in their first week of in-patient therapy at the time of inclusion. The sex of the 400 patients was male in 272 (68%) patients and female in 128 (32%) patients. Furthermore, 162 (40.5%) patients were under 60 years of age and 238 (59.5%) were over 60 years of age (Table 1). The largest diagnostic groups include 138 (34.5%) patients with head and neck cancer, 98 (24.5%) patients with rectal cancer, and 52 (13.0%) patients with lung cancer. For further information on diagnostic groups and TNM staging, see Table 1.

### 2.2. Design

At the beginning of the therapy, the patients who fulfilled the inclusion criteria were informed about the study in a personal conversation and gave their written consent. The Ethics Review Committee of the University Hospital Erlangen approved the study including the use of patients' individual data. Thus, the questionnaires could be completed directly on site and remaining questions of the patients were clarified. For the first time, the patients were interviewed during their first in-patient stay; these values determine the baseline score. For the following questionnaires, the patients were either interviewed personally at follow-up appointments or the questionnaire was sent to them by mail. They were then interviewed after six weeks, i.e., after completion of therapy, and then after each completed year after the start of therapy up to a maximum of six years or until their drop out from the study for any reason.

### 2.3. EORTC QLQ-C30 Questionnaire

The first version of the EORTC QLQ-C30 questionnaire was first published in 1987 and has been updated ever since, until the current version 3.0 was released in 1997. Since then, the questionnaire has been used in various studies around the world, giving a whole new meaning to quality of life in cancer therapy. The QLQ-C30 consists of 30 questions divided into functional scales, symptom scales, and global health status/quality of life. Functional scales are 5 multi-item scales containing physical functioning, role functioning, emotional functioning, cognitive functioning, and social functioning. Higher scores represent better quality of life. Symptom scales are 3 multi-item scales, namely, fatigue, pain, nausea and vomiting, and 6 single items such as dyspnoea, appetite loss, insomnia, constipation, diarrhoea, and financial issues. Higher scores stand for higher symptom burden causing poorer quality of life. Functional and symptom scales include the first 28 questions; answer options on a four-point scale are from 1 (not at all) to 4 (very much). The last two questions offer answer options from 1 (very poor) to 7 (excellent), resulting in the global health status and the quality of life. This questionnaire has been extensively tested for reliability and validity, making it an important instrument for measuring HRQoL of cancer patients in clinical research [29–32].

### 2.4. Statistics

The analysis was divided into three parts. First, the relationship between baseline HRQoL and OS was studied for all patients who had valid baseline values. In the second step, the relationship between HRQoL scores after therapy and OS was assessed for the same patient cohort. The third step included assessing the relationship between the change in HRQoL scores from baseline to after therapy and OS. These so-called change scores were calculated by subtracting the baseline score from the corresponding score after therapy.

The outcome variable was OS, calculated using the Kaplan-Meier method and calculated from the day of inclusion in the study until the date of death due to any cause. Univariate cox proportional hazards models (CPHM) were used to evaluate the prognostic significance of sociodemographic, clinical, and HRQoL variables [33]. In the following step, all variables that were univariate and prognostically significant were examined by multivariate CPHM for their common prognostic significance. *p* values < 0.05 were considered significant. “The minimum clinically meaningful important difference on the QLQ-C30 scales is at least 10 points” as described by Osoba et al. [34]. CPHM was used with a minimum of 10 events per variable (EPV) to increase accuracy and precision of regression coefficients and their tests of statistical significance [35, 36]. Microsoft Excel and IBM SPSS Statistics 25 were used for all calculations.

## 3. Results

The 400 participating patients were divided into two groups for each score and each time of interview according to their answers. These groups were created on the basis of the corresponding median score of all patients: group favourable HRQoL comprises the patients who in their answers reflected the better QOL, i.e., higher values on functional scales and lower values on symptom scales. The other group, also called group adverse HRQoL, represents accordingly the lower QOL, i.e., lower values on functional scales and higher values on symptom scales. The groups differ in size for each score, since one patient naturally does not receive good or bad scores throughout and not every patient answered every question (Table 2).

### 3.1. Scores prior to and after Therapy

**Figure 1:**
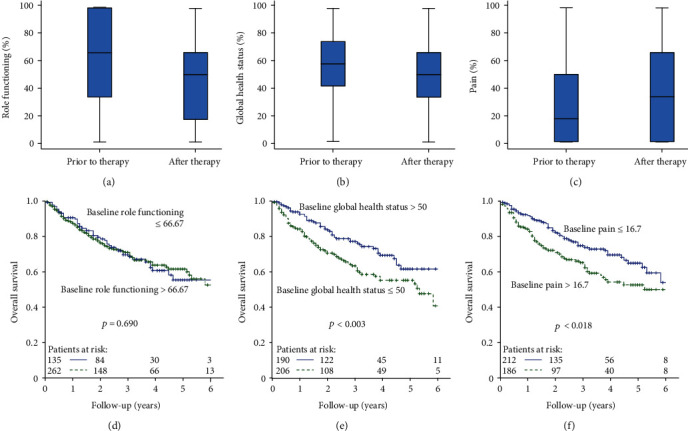
(a–c) QoL scores prior to therapy and after therapy for (a) role functioning (*n* = 239), (b) global health status (*n* = 241), and (c) pain (*n* = 244). (d–f) Baseline QoL scores and OS for (d) role functioning, (e) global health status, and (f) pain.

In order to get an initial overview of how the scores generally change in the course of therapy, box plots were created. Only coherent data were used, meaning data of patients with valid scores for both the pretreatment and posttreatment interviews. This provides a more valid representation of the changes in quality of life caused by the therapy. Role functioning and global health status are given as examples of the functional scales and pain as a representation of the symptom scales. In these box plots, the scores on functional scales decrease in the course (Figures 1(a) and 1(b)), while they increase on symptom scales (Figure 1(c)), i.e., the quality of life deteriorates overall during treatment. This applies to the median score as well as to the first and third quartiles. The median score of role functioning deteriorated from 66.7 prior to therapy to 50 after therapy with *p* < 0.0001 (Figure 1(a)), and global health status changed from 58 to 50 with *p* < 0.0001 (Figure 1(b)). Pain also deteriorated; the median score rose from 16.7 to 33.3 with *p* = 0.0001 (Figure 1(c)). This trend was found for all scales analysed. For further examples, see supplementary material, where physical functioning (supplementary figure 4a), fatigue (supplementary figure 5a), and appetite loss (supplementary figure 6a) are depicted.

### 3.2. Baseline HRQoL and Overall Survival (OS)

The two groups were then screened for their overall survival depending on their baseline score. It was observed that group favourable HRQoL survived longer than group adverse HRQoL regarding various quality of life scores.  Table 2, i-v provides the results of this analysis. Role functioning showed no relevant difference between the two groups (Figure 1(d)), whereas favourable global health status was a significant predictor of longer survival time, *p* < 0.003 (Figure 1(e)). Minor pain was also clearly associated with favourable survival, *p* < 0.018 (Figure 1(f)). Substantial differences were also observed in physical functioning (*p* < 0.001) and fatigue (*p* < 0.0005) (supplementary figure 4b and 5b). A trend was observed for emotional functioning, nausea and vomiting, appetite loss, insomnia, and obstipation, meaning the *p* value was below 0.2.

### 3.3. HRQoL Scores after Therapy and OS

**Figure 2:**
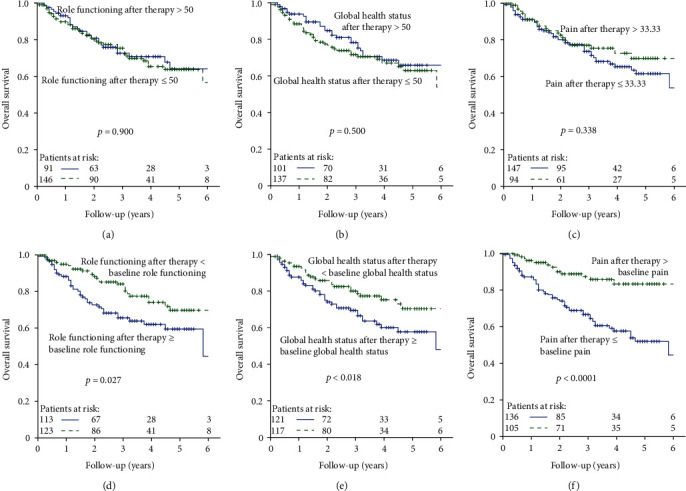
(a–c) QoL scores after therapy and OS for (a) role functioning, (b) global health status, and (c) pain. (d–f) Change scores and OS for (d) role functioning, (e) global health status, and (f) pain.

Six weeks after starting therapy, there were no significant differences between the two groups, which were also separated according to the median score (Figures 2(a)–2(c)). None of the selected HRQoL scores after therapy were predictive of survival. However, there is a trend for the adverse HRQoL group to have a shorter overall survival compared to the favourable HRQoL group. But these scores are not sufficient to make a general statement. For this reason, the change scores were further studied.

### 3.4. Change in HRQoL from Baseline and OS

The absolute difference between before and after therapy was calculated, and in a first step, two groups were formed based on the median score. The score of each patient after therapy was compared to his or her own pretreatment value.  Table 2, vi-x describes the analysis of change scores. Role functioning (*p* = 0.027), global health status (*p* < 0.018), and pain (*p* < 0.0001) were predictive of survival (Figures 2(d)–2(f)). Patients whose global health status and role functioning scores after therapy were worse than prior to therapy or who had more pain survived clearly longer than the other group. In the categories fatigue, appetite loss, and insomnia, the adverse HRQoL groups tended to a better outcome (supplementary Figures 5e, 6e).

**Figure 3:**
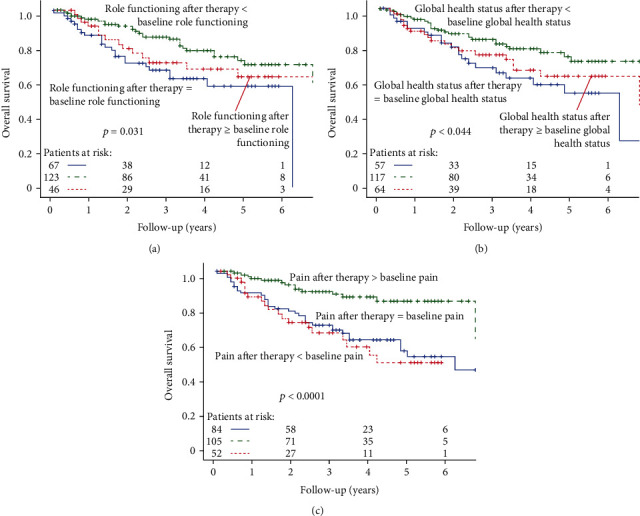
(a–c) Change scores and OS for (a) role functioning, (b) global health status, and (c) pain regarding three groups.

In a second step, three groups were created: the “no change” group contains patients whose scores did not change in the course of therapy, “adverse” group contains those patients whose scores after therapy were worse than before, and “improved” group comprises patients whose scores improved compared to prior to therapy (Figure 3). Regarding role functioning, the adverse group survived clearly longer than the other two groups, *p* = 0.031. Adverse global health status and pain were also distinct predictors of improved survival with *p* = 0.044 and *p* < 0.0001, respectively.

### 3.5. Univariate and Multivariate Analyses of Baseline Scores and Prognostic Factors for OS

To rule out any potential confounding, baseline QoL scores were added to the univariate and multivariate analyses. Well-known prognostic factors such as TNM classification, age, or gender were compared to different baseline QoL scores. The QoL scores were previously selected by significant Kaplan-Meier estimates. QoL scores then included global health status, functional scores, such as physical functioning and role functioning, and symptom scores such as fatigue, pain, and appetite loss.  Table 3 describes the univariate and multivariate analyses of the baseline scores.

The univariate analysis reveals that M status and age were significant predictors of survival. Pain was borderline significant (*p* = 0.054). Multivariate analysis indicates that high global health status (*p* = 0.005) and mild pain (*p* = 0.04) were favourable for survival. Young age and M0 category were also beneficial. Patients with low global health status at baseline have a 1.76 times higher risk of dying (HR = 1.758). Moreover, patients who experienced more pain prior to therapy had a 1.5 times higher risk of dying (HR = 1.489).

### 3.6. Univariate and Multivariate Analyses of Change Scores and Prognostic Factors for OS

Change scores were also subjected to univariate and multivariate analyses in order to account for confounding.  Table 4 describes the changes in QoL scores from baseline to after therapy. TNM staging, age, and gender were compared to physical functioning, role functioning, global health status, fatigue, pain, and appetite loss. Regarding univariate analysis of all analysed QoL scores, only mild pain was beneficial for survival (*p* = 0.002). Another favourable predictor of survival was young patient age, *p* = 0.023. Concerning multivariate analysis, pain remained significant (*p* = 0.001). Further significant variables were the patient age and M category with *p* = 0.009 and *p* = 0.029, respectively. Pain was the superior predictor of survival of all variables and QoL scores studied. A deterioration in pain was associated with a 2.8 times higher change of longer survival (HR 0.36).

## 4. Discussion

It is generally assumed that patients whose quality of life deteriorates in the course of cancer therapy compared to the past have a worse outcome and thus a lower survival rate. Our aim was to find out whether these patients really do survive less than patients whose quality of life either does not change or even improves over the course of therapy. Therefore, we analysed the scores prior to therapy, after therapy, and the difference between these two points in time, creating 2 and 3 groups.

Our data confirm other studies that the baseline score is a meaningful predictor of survival. We were able to demonstrate prognostic relevance for physical functioning, global health status, fatigue, and pain at baseline. In addition, the change score is also appropriate to make a statement about the survival of cancer patients. Various studies have already shown that an improvement in HRQoL in the course of therapy leads to longer survival [24, 37, 38]. We could prove exactly the opposite, i.e., that patients who deteriorate in their quality of life during the course of therapy survive longer than those who showed self-reported improvement in quality of life. We were able to demonstrate significant prognostic relevance for the change scores of role functioning, pain, and global health. These counterintuitive findings have also been found in other clinical trials. Oskam et al. reported that a deterioration of global quality of life after treatment is a predictor of survival in patients with oral or oropharyngeal cancer [28]. Braun et al. showed that an improvement in social function at 3 months is associated with worse survival [25]. Ediebah et al. reported a higher risk of death for patients with a self-reported improvement over time for functional scales and global health status [21]. A deterioration of the quality of life could be responsible for an increased response to the therapy, especially in normal tissue. The cause could be an increased sensitivity of the patients to radiation [39], which leads to increased side effects and at the same time to an enhanced effect on the tumour cells.

## 5. Conclusion

The general assumption that patients whose quality of life and symptom burden worsens in the course of therapy ultimately die earlier than patients whose quality of life remains the same or improves must be reconsidered. The natural resilience and coping strategies of patients with poorer quality of life appear to be underestimated. Not only the baseline HRQoL is a predictor of survival but also the change due to therapy, more specifically the worsening. The decisive factor is not only the quality of life immediately after therapy but also the change compared to before. This is a new prognostic factor regarding the survival of patients receiving radiochemotherapy. These results naturally raise new questions that could decisively influence and change the therapy. For example, it should be investigated whether patients with a worsening condition under therapy receive higher doses of chemotherapy or more intensive radiation, or whether they respond more intensively to the therapy individually and thus take all side effects with them.

In conclusion, HRQoL should be recorded continuously in the course of therapy and the results of the surveys should be included in the therapy. It might be possible to find out which type of therapy is related to changes in quality of life and how. In addition, early deterioration of patients can be detected and interventions can be planned that potentially improve both HRQoL and outcome. Nevertheless, distortive effects should be considered and excluded, as change score analyses can be biased by floor and ceiling effects at baseline [21].

## Figures and Tables

**Table 1:** Patient demographics.

		*n*	(%)
Gender			
Male		272	(68)
Female		128	(32)
Age (yrs)			
<60		162	(40.5)
>60		238	(59.5)
Diagnostic group			
Head and neck		138	(34.5)
Rectal		98	(24.5)
Lung		52	(13)
Gastrointestinal		34	(8.5)
Gynaecological		21	(5.3)
Others		57	(14.2)
TNM staging			
T	0	16	(4.0)
	1	51	(12.7)
	2	92	(23.0)
	3	136	(34.0)
	4	105	(26.3)
N	0	147	(36.7)
	1	253	(63.3)
M	0	322	(80.5)
	1	78	(19.5)

**Table 2:** Analysis of QoL scores. Figures quoted in parts i-v describe baseline scores. First value: group favourable HRQoL (higher QOL)/second value: group adverse HRQoL (lower QOL). The median scores were used as cut-off-points. Figures quoted in parts vi-x describe change scores. First value: group favourable HRQoL (better scores after therapy than baseline)/second value: group adverse HRQoL (equal or worse scores after therapy than baseline).

	Baseline score					Change score				
	i	ii	iii	iv	v	vi	vii	viii	ix	x
	Median score (QoL)	*n*	Median OS (months)	4-year OS (%)	Log-rank *p* value	Median score (QoL)	*n*	Median OS (months)	4-year OS (%)	Log- rank *p* value
Functional scales										
Physical functioning	93.3/60.0	168/232	<80/63	71.4/54.9	<0.001	73.3/86.7	100/143	<76/-	67.5/65	0.400
Role functioning	100/33.3	135/262	<76/<84	60.6/63.6	0.690	50.0/66.7	113/123	<70/<80	>59.1/74.2	0.027
Emotional functioning	83.3/41.7	189/208	70-310/64	67.0/57.7	0.122	58.3/75.0	138/86	-/70	>61/66.5	0.700
Cognitive functioning	100/66.7	178/219	76/70	64.9/60.2	0.560	83.3/83.3	161/79	76/52-310	70.1/64.9	0.820
Social functioning	100/50.0	130/267	-/76	63.2/62.1	0.900	50.0/66.7	131/92	70/<80	65.2/>62.8	0.600
Global health status	66.67/33.3	190/206	80-310/63	68.9/55.6	**<**0.003	50.0/66.7	121/117	<70/<310	60.7/75.5	0.018
Symptom scales										
Fatigue	22.2/66.7	237/163	76/<63	68.5/52.8	<0.0001	44.4/33.3	110/132	70/<310	>62.5/70.7	0.137
Pain	0/50.0	212/186	<76/63	69.6/54.4	<0.018	33.3/0	136/105	<70/-	57.3/83.5	**<**0.0001
Appetite loss	0/66.7	224/174	<70/<76	66.9/55.7	0.102	0/0	138/98	<70/76-310	63.5/74.3	0.113
Insomnia	0/66.7	265/130	80/63	66.4/52.8	0.103	33.3/0	167/70	<76/<310	65.5/<76.3	0.142
Nausea and vomiting	0/33.3	300/98	<76/63	64.3/54.8	0.198	0/0	137/87	76/62	68.2/>55.9	0.189
Dyspnoea	0/33.3	209/186	-/<70	62.0/>60.7	0.450	0/0	170/53	<76/62-80	67.2/>51.9	0.290
Constipation	0/33.3	264/133	<80/<76	64.1/>56.7	0.090	0/0	141/82	76/-	65.3/>58.8	0.420
Diarrhoea	0/33.3	267/128	<76/70	58.1/>68.5	0.140	0/0	160/78	<76/-	67.4/70.2	0.220
Financial difficulties	0/66.7	221/172	<70/<310	61.1/64.5	0.114	0/0	185/36	76/-	65.9/<71.6	0.560

**Table 3:** Univariate and multivariate analyses according to Cox's proportional hazards model for sociodemographic and clinical variables and HRQoL scores at baseline.

	Univariate analysis			Multivariate analysis		
	Hazard ratio	95% C.I.	*p*	Hazard ratio	95% C.I.	*p*
T category (T1/T2 (*n* = 137) vs. T3/T4 (*n* = 223))	1.141	0.743–1.753	0.547	—	—	—
N category (N0 (*n* = 131) vs. N+ (*n* = 229))	1.351	0.857–2.13	0.195	1.415	0.918–2.182	0.116
M category (M0 (*n* = 303) vs. M+ (*n* = 57))	2.334	1.471–3.703	0.0003	2.743	1.829–4.144	**<**0.0001
Gender (male (*n* = 249) vs. female (*n* = 111))	1.096	0.724–1.661	0.665	—	—	—
Age (<60 years (*n* = 149) vs. >60 years (*n* = 211))	1.887	1.248–2.853	0.003	2.059	1.379–3.072	0.0004
Physical functioning (above (*n* = 161) vs. below median (*n* = 199))	1.533	0.932–2.521	0.093	1.299	0.835–2.019	0.246
Role functioning (above (*n* = 125] vs. below median (*n* = 235))	0.774	0.497–1.206	0.258	—	—	—
Global health status/QoL (above (*n* = 176) vs. below median (*n* = 184))	1.481	0.947–2.315	0.085	1.758	1.184–2.613	0.005
Fatigue (below (*n* = 221) vs. above median (*n* = 139))	1.091	0.676–1.762	0.722	—	—	—
Pain (below (*n* = 193) vs. above median (*n* = 167))	1.518	0.993–2.323	0.054	1.489	1.018–2.178	0.040
Appetite loss (below (*n* = 205) vs. above median (*n* = 155))	0.855	0.55–1.328	0.485	—	—	—

**Table 4:** Univariate and multivariate analyses according to Cox's proportional hazards model for sociodemographic and clinical variables and HRQoL change scores.

	Univariate analysis			Multivariate analysis		
	Hazard ratio	95% C.I.	*p*	Hazard ratio	95% C.I.	*p*
T category (T1/T2 (*n* = 78] vs. T3/T4 (*n* = 138))	1.144	0.629–2.081	0.660	—	—	—
N category (N0 (*n* = 77) vs. N+ (*n* = 139))	1.290	0.694–2.397	0.421	—	—	—
M category (M0 (*n* = 183) vs. M+ (*n* = 33))	1.745	0.956–3.187	0.070	1.895	1.069–3.359	0.029
Gender (male (*n* = 148) vs. female (*n* = 68))	1.068	0.607–1.879	0.820	—	—	—
Age (<60 years (*n* = 93] vs. >60 years (*n* = 123))	1.954	1.095–3.454	0.023	2.113	1.209–3.695	0.009
Physical functioning (above (*n* = 92) vs. below median (n = 124))	1.742	0.904–3.355	0.097	1.487	0.832–2.657	0.180
Role functioning (below (*n* = 103) vs. above median (*n* = 113))	0.712	0.391–1.297	0.267	0.61	0.359–1.039	0.069
Global health status (above (*n* = 113) vs. below median (*n* = 103))	0.555	0.298–1.034	0.064	—	—	—
Fatigue (above (*n* = 100) vs. below median (*n* = 116))	0.927	0.489–1.757	0.816	—	—	—
Pain (below (n = 119) vs. above median (*n* = 97))	0.356	0.184–0.688	0.002	0.36	0.193–0.67	0.001
Appetite loss (below (*n* = 125) vs. above median (*n* = 91))	0.783	0.431–1.424	0.423	—	—	—

## Data Availability

The datasets analysed in this study are available on request from the corresponding author.

## Abbreviations

CPHM: Cox proportional hazards models
EORTC: European Organisation for Research and Treatment of Cancer
HRQoL: Health-related quality of life
OS: Overall survival
QoL: Quality of life.

## Supplementary Materials

Supplementary Materials Additional figures showing the results of the analysis of other QoL scores and their correlation with OS. Supplementary Figure 4: physical functioning; scores prior to and after therapy (a), baseline scores and OS (b), scores after therapy and OS (c), change scores and OS in 3 groups (d), and change scores and OS in 2 groups (e). Supplementary Figure 5: fatigue; scores prior to and after therapy (a), baseline scores and OS (b), scores after therapy and OS (c), change scores and OS in 3 groups (d), and change scores and OS in 2 groups (e). Supplementary Figure 6: appetite loss; scores prior to and after therapy (a), baseline scores and OS (b), scores after therapy and OS (c), change scores and OS in 3 groups (d), and change scores and OS in 2 groups (e).
