# Supplementary material for: Deterioration of Health-Related Quality of Life Scores under Treatment Predicts Longer Survival
Source: Biomed Res Int. 2020 Aug 17;2020:3565238. doi: 10.1155/2020/3565238 (PMC7448240; doi:10.1155/2020/3565238)
Supplement: Supplementary Materials — Additional figures showing the results of the analysis of other QoL scores and their correlation with OS. Supplementary Figure 4: physical functioning; scores prior to and after therapy (a), baseline scores and OS (b), scores after therapy and OS (c), change scores and OS in 3 groups (d), and change scores and OS in 2 groups (e). Supplementary Figure 5: fatigue; scores prior to and after therapy (a), baseline scores and OS (b), scores after therapy and OS (c), change scores and OS in 3 groups (d), and change scores and OS in 2 groups (e). Supplementary Figure 6: appetite loss; scores prior to and after therapy (a), baseline scores and OS (b), scores after therapy and OS (c), change scores and OS in 3 groups (d), and change scores and OS in 2 groups (e). [file 3565238.f1.pdf]

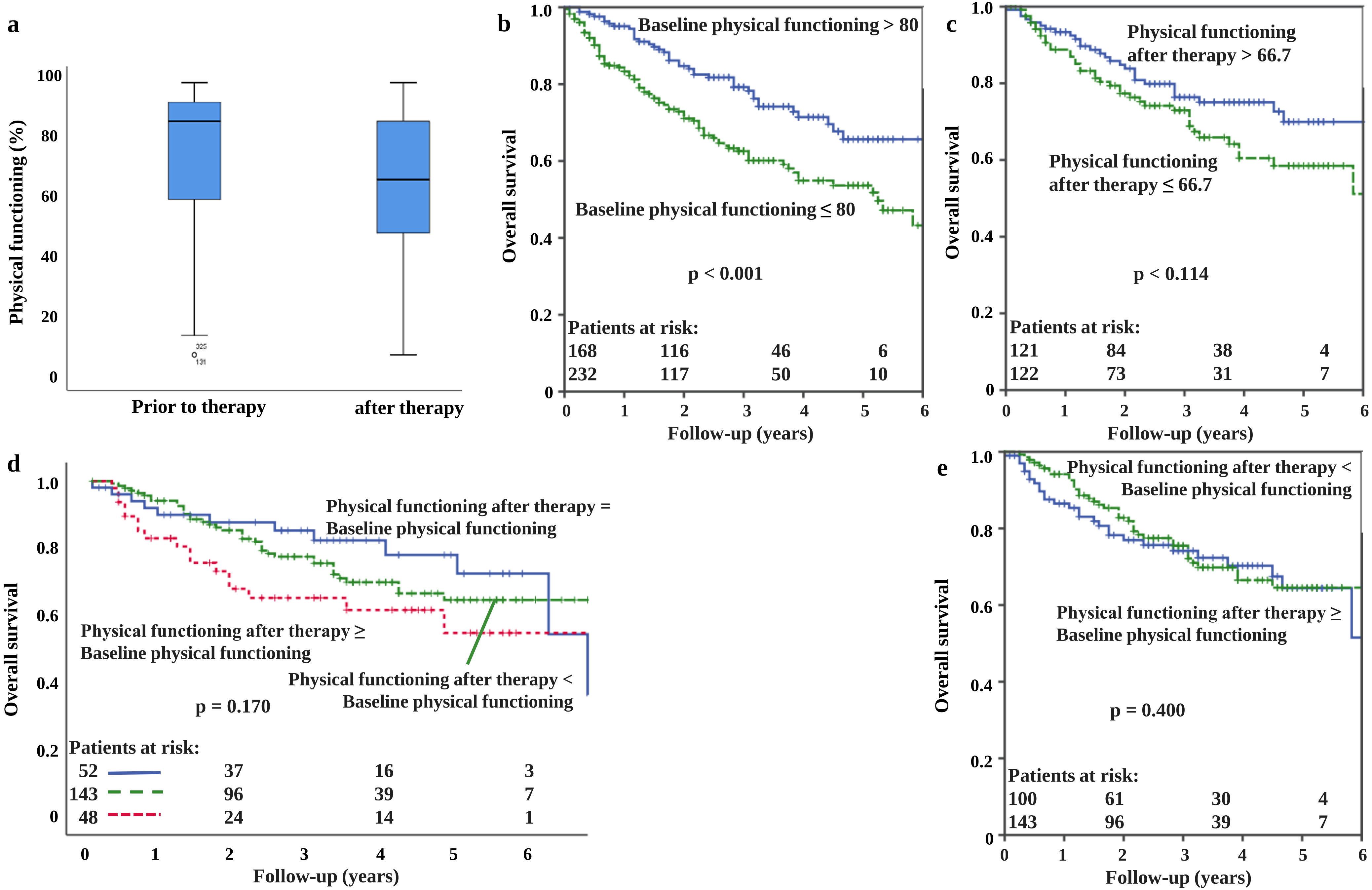

Supplementary figure 4: Physical functioning; scores prior to and after therapy (a), baseline scores and OS (b), scores after therapy and OS (c), change scores and OS in 3 groups (d), change scores and OS in 2 groups (e).

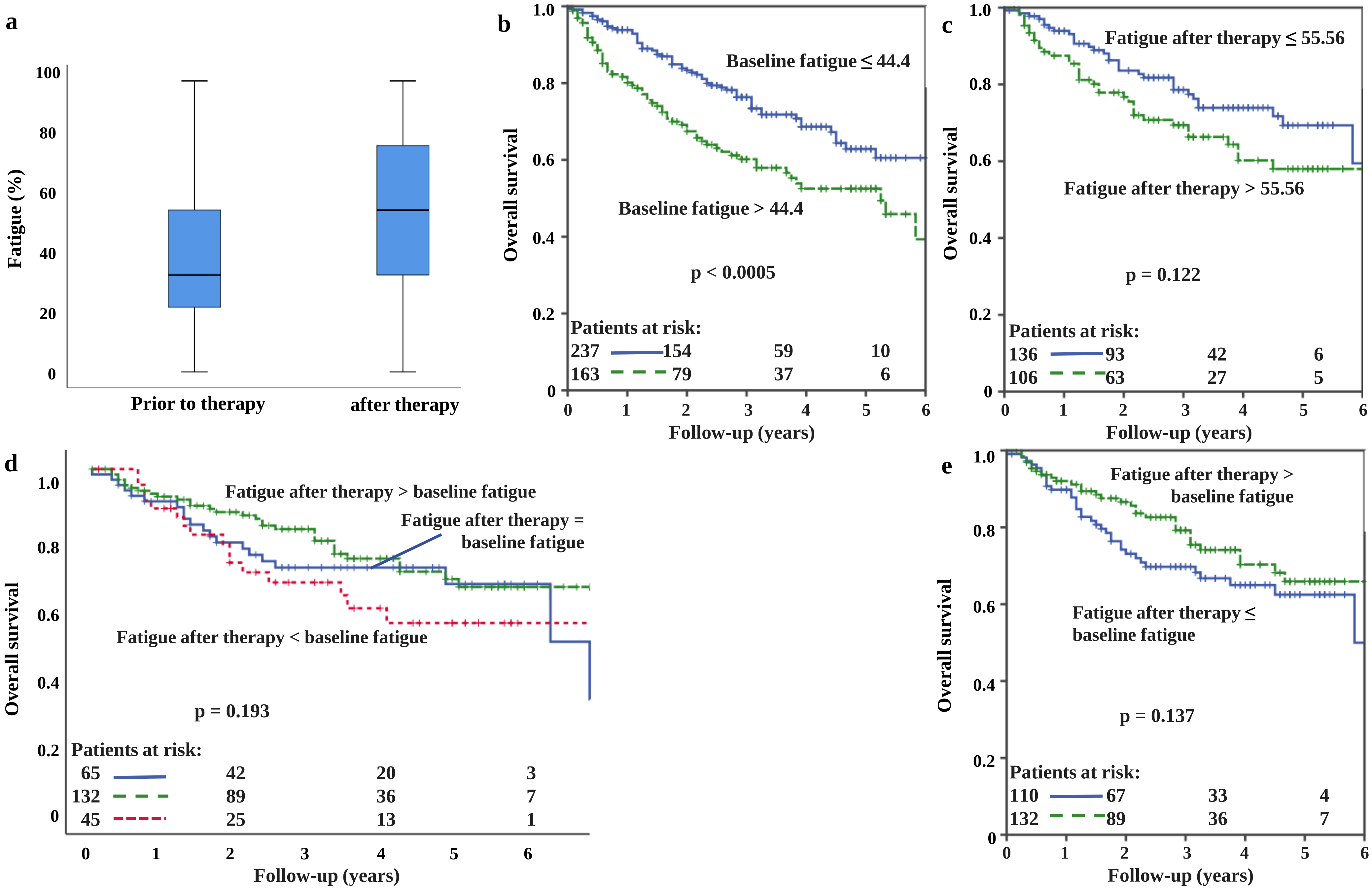

Supplementary figure 5: Fatigue; scores prior to and after therapy (a), baseline scores and OS (b), scores after therapy and OS (c), change scores and OS in 3 groups (d), change scores and OS in 2 groups (e).

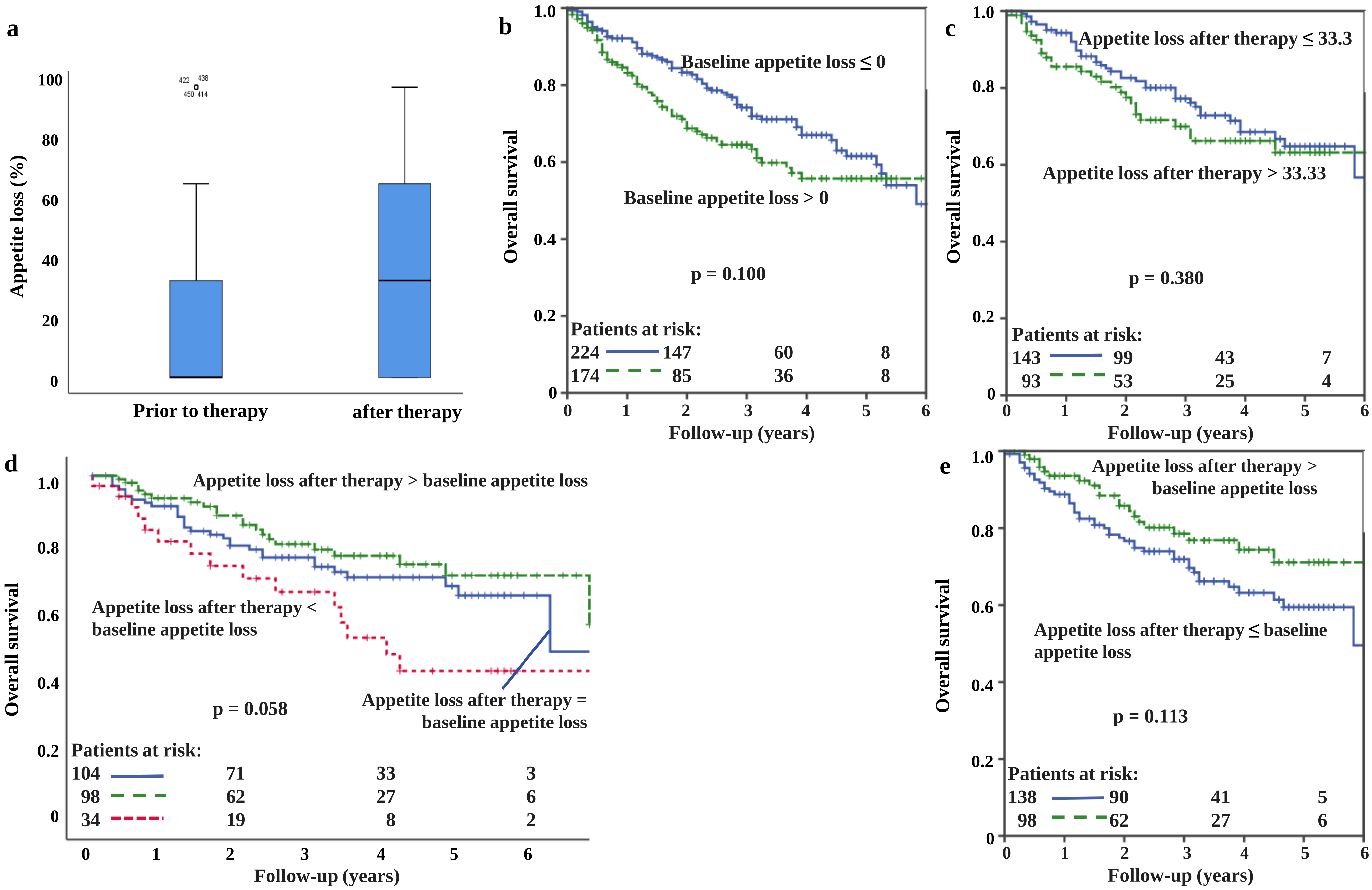

Supplementary figure 6: Appetite loss; scores prior to and after therapy (a), baseline scores and OS (b), scores after therapy and OS (c), change scores and OS in 3 groups (d), change scores and OS in 2 groups (e).
